# Address, Introductory to the Sixteenth Annual Course of Lectures in Rush Medical College

**Published:** 1858-11

**Authors:** J. W. Freer


ARTICLE II.

### ADDRESS, INTRODUCTORY TO THE SIXTEENTH ANNUAL COURSE OF LECTURES IN RUSH MEDICAL COLLEGE:

DELIVERED NOVEMBER 1, 1858.

BY J. W. FREER, M. D.

GENTLEMEN OF THE MEDICAL CLASS:—The labors of the sixteenth session are at hand. By the kind courtesy of my colleagues, it becomes my agreeable duty to welcome you to the halls of Rush Medical College.

I see before me those whose faces are familiar, from former associations in this institution, and whose return indicates their satisfaction with our labors in the past.

With others an acquaintance is yet unformed; but your presence to-night, gentlemen, is our best assurance of your devotion to the honorable profession of medicine, and of your desire to fit yourselves for honorable positions in its practice.

Permit me then, in behalf of the Faculty, to extend to you the hand of fraternal regard, and welcome you to the labors and the rewards of the session.

We desire to greet you as diligent students, thirsting for that knowledge of the profession of your adoption, which is indispensable to success, and shall spare no efforts on our part to render our instructions lucid and practical, and, so far as the brief time will permit, prepare you for future usefulness.

Common pursuit induces common sympathy; and, in proportion as the powers of the human mind are taxed in the attainment of a great object, so are minds thus laboring more closely drawn together by common sentiment, till those who may have met as strangers part with reluctance; and if your industry shall realize our hopes, we shall rejoice at your advancement, but sorrow at your parting.

In making the profession of medicine your choice, we sincerely hope you have not underrated the labor incident to success.

It has no offers of inglorious ease with which to lure its votaries. It does, indeed, strew beautiful flowers in the pathway of its followers, but spreads no wayside couch for idle dreamers.

Its pathway is not over level plains that one may tread with ease, but rugged Alps are in its course, and he who highest climbs, may still behold a broad expanse where student's footprint never yet was seen.

But though the goal seems hidden in the clouds, the rewards of labor lie all along the pathway; and, as new truths in medicine are unfolded to your view, your severest labor will become at last your greatest pleasure.

The true mission of medicine is only appreciated by the reflecting mind. It has to do, not with the earthly treasures of man, but stands as the handmaid of nature, to strengthen the ever-failing tie which binds his mortal to his immortal being.

With it are the issues of life and death, and tributary to its use, are all of the important sciences.

If life be dear, or health valuable, then must we cherish that science which shall secure the one and preserve the other.

The beauty of the human system is only equaled by its perfection. It came from the hands of its Creator, the climax of his works! but, from the time the mandate was issued, "to dust shall thou return," disease has ever lingered near, ready ot accomplish that decree, if any point be left unguarded.

How then shall we, as the conservators of health, be able to guard this human citadel without a knowledge of the structure and strength of the fabric; its portals and its avenues—its chemical laboratories—its myriad of work-houses, where repairs are ever going on; its telegraphic structures, communicating with all parts, and conveying signals with the swiftness and precision of lightning; and even its conduits and its cesspools, through which it is relieved of worn-out and useless matter.

This complicated structure, it is the province of scientific medicine to protect; and in order to succeed, we must trust, not alone to armory, but must know well our battle-ground. In a word, gentlemen, know all that may be known of the anatomy of the human *system*.

A thorough knowledge of the structures of the body, and their functions in *health*, can alone fit you to recognize those departures which constitute disease.

This instruction it is the united province of anatomy and physiology to *impart*.

Nor is the science of pathology less important. It traces morbid results back to their essential cause. With what unerring precision does it point to the rapid pulse; the parched tongue; the burning skin; the hurried breath; the anxious eye; the stifled groan; and interpret the signals of distress which tell of the fearful conflict waged within. It hears alike the *thrill*, the *murmur*, and the *rale*, and notes their tale of woe. It stands our great interpreter of the signs, symptoms and results, which constitute the *language of disease*. Cost what it may of time or trouble—that language you must understand.

But mark the *lesion;* note its accurate pathology.

Still your labors end not here. Remedial agents, your weapons for this warfare, are yet to be *discovered,* and the method of their administration made known. No single locality can supply these varied wants. They may lie in profusion upon the surface, or deep in the bosom of the earth. With one hand you may cull them from the herbage of the pathside, with the other be compelled to gather them from the distant sea-girt isle.

They rise from their resting places *not* with the label of their virtues impressed upon them! These they discover only to the careful analyst and faithful experimenter.

The chemist's eye has never yet discovered the confines of this vast field. Yet the single department of Materia Medica claims this whole domain.

When you shall be able to tell the names and relations of each artery, vein and nerve—each bone and muscle—each organ, with its functions, its characteristic appearances in health, the changes induced by disease—the virtues of the plants upon the thousand hills—the properties of the minerals and the metals—the incompatibles in medicine—the wonderful story of minute structure which the microscope reveals—and the modifying influences of age, sex, temperament, climate and hereditary disease upon the human system; *then,* only, would you be able to answer with appropriate emphasis whether in all your labors you had found repose.

How boundless are these fields of study! Your tears will never flow for unconquered realms. There are no weeping Alexanders in the medical domain.

Should you devote the energies of your lives to chemistry alone, I doubt not Baron Liebeg might still be your instructor. Should botany beguile your hours, still I fancy some Pereira might lead you all your lives.

That renowned surgeon, who hardly failed to prosecute dissections a single day for forty years, still expressed that he had much to learn. If I point you to the surgeons of renown in this or other lands, they are all, without exception, enthusiasts in their departments. Nothing but severe and long-continued labor in their particular field has rendered any of our *worthies* pre-eminent. That man has never lived who stood prominent in all departments. It can only be your aim to equal *one*—not all of these—if your ambition leads you to seek for fame in unexplored fields.

If your studies had only reference to *yourselves*, we would advise you to glance at the more prominent and important *truths* in medicine; thence turn your energies to that department most in harmony with your tastes, and there seek your preferment in original investigations.

But we cannot so regard those who listen to us to-night.

The preservation of health, and the cure of disease, according to the prescribed rules of practice, for the most part, are the objects of your future anticipations.

We remember that but a few months at most intervene between this pleasant interview and the hour when the weighty responsibilities of your mission will rest heavily on you—when the writhing victim of disease shall turn to you the imploring cry for help—when each word and look of yours shall wing its way with joy or sorrow to the hearts of stricken friends.

And, when you stand thus single-handed in the conflict of life and death, it will avail much that you are well informed in all of the auxiliary sciences of medicine.

Yet, if the labors of a life could be devoted to each of these departments, and their united results were at your command, you would be only the better prepared to accomplish your desire.

We perceive that, in assuming the duties of practitioners of medicine, we are not realizing the earthly heaven of indolence.

He who shuns the labor incident to success in agriculture, or the various trades, thinking to become a competent physician, and yet gratify lazy habits, never committed a more egregious blunder; and the sooner he discovers his mistake the better for himself and mankind.

We trust that the teachings of this institution, in all its past history, will deter any such from entering here.

We are proud to number among the alumni of Rush Medical College those who are foremost to sustain and advance the interests of scientific medicine; and are unwilling to believe the present class less anxious to do honor to themselves and their prospective "alma mater" than their predecessors.

Gentlemen,—although we have dwelt thus long on the difficulties and obstacles besetting the path of the medical student, yet it has not been our purpose to discourage you from entering this great and noble field; nor do we deem it necessary to warn you against indolence, but rather seek to introduce the obstacles in your way, and, as they are comprehended, you will be the better prepared to appreciate advice which, if faithfully followed, will lead to ultimate success.

The great question which most concerns the medical student is this: how to select from the great mass of medical lore those portions which will soonest fit him for usefulness; and it is one of the special duties of the institution to guide and direct the student in the attainment of this important object. But we cannot expect in the brief time alloted us, to render you profoundly versed in any one of the departments of scientific medicine. We cannot expect more than to be able to present to your notice the indispensable portions, thereby enabling you during the period of pupilage to attain sufficient practical knowledge to pursue the healing art in the ordinary acceptation.

We hope there are those among you who aspire to something more than the title of "ordinary" practitioners of medicine; but experience teaches us that about nine-tenths of the graduates from the various medical schools come to this at last. We do not wish to be understood as underrating this nine-tenths, for they go to constitute the most useful class of persons in the world. To borrow a phrase from a distinguished southern politcian, they are the very "mud sills" of medical society, if not of medical science.

We hope there are embryo Sir Astley Coopers among us, and perhaps there are; but it will require a longer period of gestation than is natural to this or any similar institution, to bring the promising "chick" to full development.

Great men are not furnished to order by colleges; but those institutions may show the way to preferment. In other words, the medical student may look upon his college days as a period of incubation, with this difference, that it depends very much on himself whether he turns out addle or not.

Again, let me assure you that the time allowed for the acquirement of that which is indispensable in ordinary practice is barely sufficient for your necessities, and will bear no diminution for bestowment on extraneous objects.

A thorough knowledge of our mother tongue is indispensable to the medical student. Indeed, no person should be admitted to membership with a medical class without this necessary qualification; and if there are those among you who have it not, we would advise you to retire until this needful task is accomplished, otherwise you must eventually submit to more humiliating positions than "mud sills," even that of poor relations of that very substantial class.

It is of great advantage to have a knowledge of all the modern languages, especially the German and French, for we get the spirit and meaning of an author more readily when expressed in his native tongue; and indeed, foreigners are becoming so numerous with us, that it has become almost a necessity that the medical practitioner should be capable of conversing in various languages. But if you have not already gained these needful accomplishments, it will have to be deferred until a more convenient time.

The means, then, which shall render you good physicians and competent surgeons must engage your chief attention during the period of pupilage.

To amateur students, and experts, must we look in the main for extended investigations in the several sciences allied to medicine, and in their departments we must regard them as benefactors. But these extended investigations can never be accomplished by the general practitioner; his limited time absolutely forbids it. He must be content in the main to seize upon and turn to good account the researches of others.

The teachings of some of the medical schools, I think, have been erroneous in this respect; and perhaps we are not without blame. It is too often forgotten that the medical student is soon to become the medical practitioner, and his mind is lumbered too much with the technical legerdemain of test tubes and microscopes.

"It should be constantly borne in mind that, when you come to treat disease, you approach the bedside as physicians and surgeons, and not merely as chemists, anatomists or botanists. Such is the character in which you are to appear, and the acquisition of knowledge which will prepare you for the discharge of its duties, ought to engage your chief attention."

The collateral branches of medicine must themselves be advanced, and their connection with the latter in its aspect as an art gradually evolved. But it is rather the chemist that will extend chemistry; the naturalist botany; and the comparative anatomist will effect what is necessary for his own department. These things cannot be done by the ordinary practitioner of medicine, and he must, as the rule, be contented to leave to the few greater intellects of the time even the duty of developing the relations borne by each progressive step made in the above departments of knowledge to the principles and utilities of his own profession.

That there is now a fashion leading to opposite doctrines than these, we are fully aware. We see it operating far too frequently, both upon student and upon teacher. With the schools there is much tendency to progress—much improvement in many points, yet how much error! Too often one would think it is quite forgotten that the medical student is soon to become the medical practitioner.

In anatomy, practical and human, we hear of more than half the session being consumed with "homologies" and developing mental and transcendental anatomy of the bones, being so learnedly and fully accompanied by comparative illustrations, that three months barely suffice for their consideration, though ample enough to cause the vessels and nerves to be neglected.

In physiology, we have learned disquisitions upon "caudate cells," epithelial scales and basement membrane, absorbing the time that should be spent in the more critical examination of the functions of the different organs.

It is well to know of nuclei and nucleoli, and to understand the cell theory; but it seems more important, if you must neglect one or the other, that you should know the course of and parts contiguous to a large artery.

Nor are the teachings of chemistry, as too often pursued in the schools, without fault in this respect. The minutiæ and subtleties of organic analysis, the formulas and the symbols of theoretic combinations, are all very well for the accomplished chemist; but there is more of utility to the practitioner in understanding the principles of ventilation, the injurious effects of organic decomposition, the action of lead upon water, of copper on food, or the effect of adulteration of articles of daily consumption, by the sick and well.

In a word, gentlemen, I hold to practical utility in medical teaching, and let scientific disquisitions take care of themselves. I believe that instead of theorizing too much, you had better be engaged in educating your eyes, your ears, and your touch, that you might become competent to distinguish, with your own eyes, the difference between scarlatina and measles. Your own ear should distinguish between mucous and crepitant rales; your own touch, the peculiar differences of the pulse in enteric inflammation and exhausting hemorrhage.

The bedside of the patient is the stand point where you may apply the teachings of the lecture room. It is there you may learn the obstructed respiration of pneumonia—the prostration of peritonitis—the laryngial breathing of croup—the symptoms of cardial derangement in rheumatism—the symptoms of internal hemorrhage—the vomiting of constricted hernia—the hiccough of gangrene—and the collapse of intestinal perforation.

Hence the necessity of hospital instruction in connection with and illustration of daily lectures. In the lectures you hear the statement of facts; in the clinical instruction you behold them verified.

Nor are you to suppose that our largest institutions afford the greatest facilities for hospital instruction. Indeed the reverse is true where ample hospital advantages are furnished; for the larger the class, the more must clinical instruction be addressed to the mass, and less to individuals. Often the number in attendance is so great, that the mass of observers are so far removed from the patient that a critical examination is impossible, and deliberate inspection out of the question.

With the hospital advantages connected with this school, we are confident, you will find our facilities for benefiting you in that respect far more practical than though your number was twice as great.

We come now to answer the question, How shall you proceed in order to gain the requisite knowledge for the practice of our profession in the brief time alloted for its acquisition?

In the first place, give diligent heed to the instruction of those who, in a brief course, may impart to you the result of years of experience and labor in their departments; at the same time, I warn you against being blind recipients of whatever may be offered by your instructors without canvassing its merits. At this early period you should commence reasoning for yourselves; take no man's word as to the truth of a dogma or hypothesis, before submiting it to your own mental crucible.

The blind follower of others' doctrines in medical science is merely a receptacle of all the truths and errors that may chance to come in his way. Minds thus constituted are not fit for the high calling of physicians, but should assume the menial offices for which by nature they are adapted, and in which, by following the dictum of more gifted independent minds, they may become really useful.

In the second place, learn to be close and careful observers at the bedside, in the hospitals, until you are able with your own five senses to distinguish between the different diseases presented for your inspection. I tell you, gentlemen, there is nothing of greater advantage to a young man in practice, than to be able at a glance to tell the "old ladies what the matter is." Indeed, once I knew of a poor doctor who was ruined for want of this qualification; for he mistook a case of parturition for that of bilious fever, and proceeding to put up his powders, told the officiating *beldame* that he had a man just in that fix the day before, and *cured* him at once.

Again, in regard to first course students, I would recommend that you bend your minds principally to the fundamental sciences, embracing anatomy, physiology, chemistry and materia medica

—especially anatomy, the basis and foundation of the whole superstructure of scientific medicine. Without it, you might as well undertake to master a language without the alphabet. To it, the heroes of our profession owe their preferment.

It follows, then, that the anatomy of the human system, with the functions of the different organs, should engage the chief attention of the junior members of the class. Then you will be prepared, during your second course, to comprehend the teachings and doctrines of medical and surgical practice. And let the few months spent here in study be so improved that in after life you may recall the time with pleasurable remembrances of profit and enjoyment.

Shun, as associates, those who fail to appreciate their advantages; for although they may seem careless and happy, yet the time will surely come when they will regret, in sackcloth and ashes, the misspent time of their college life.

Carefully avoid those temptations and excesses which have ruined the hopes and expectations of so many of our young men.

Remember that you have assumed a position where the world has a claim on you for your best services. It may rightfully demand of you a well disciplined mind, a body unimpaired by excesses, and a heart pure and philanthropic. Seek those attainments which will render you the desirable companions of the leading minds which stand as lights in the profession, and which will render you ministering angels to those who turn in deepest sorrow, and commend to your skill the wellbeing of dearest friends. Above all, fit yourselves so as to be able to practice our profession with an approving conscience; with that self-reliance and manliness that conscious ability can alone impart; and that in your declining years you may repose on the grateful remembrance that those who have committed the greatest treasures to your care, have not been the "victims of misplaced confidence."

For the education of such men this institution was projected, and to the honor and credit of its founder, it has not as yet materially failed in its object.

